# Cysteine Donor-Based Brain-Targeting Prodrug: Opportunities and Challenges

**DOI:** 10.1155/2022/4834117

**Published:** 2022-02-24

**Authors:** Gaoyang Ni, Zhenbiao Hu, Ziteng Wang, Min Zhang, Xingyu Liu, Guihong Yang, Zhaowei Yan, Yang Zhang

**Affiliations:** ^1^School of Biology and Food Engineering, Changshu Institute of Technology, Changshu, 215500 Jiangsu, China; ^2^Key Laboratory of Structure-Based Drug Design & Discovery, Ministry of Education, Shenyang Pharmaceutical University, Shenyang, 110016 Liaoning, China; ^3^Department of Pharmacy, The First Affiliated Hospital of Soochow University, Suzhou, 215006 Jiangsu, China; ^4^College of Pharmaceutical Sciences, Soochow University, Suzhou, 215123 Jiangsu, China

## Abstract

Overcoming blood-brain barrier (BBB) to improve brain bioavailability of therapeutic drug remains an ongoing concern. Prodrug is one of the most reliable approaches for delivering agents with low-level BBB permeability into the brain. The well-known antioxidant capacities of cysteine (Cys) and its vital role in glutathione (GSH) synthesis indicate that Cys-based prodrug could potentiate therapeutic drugs against oxidative stress-related neurodegenerative disorders. Moreover, prodrug with Cys moiety could be recognized by the excitatory amino acid transporter 3 (EAAT3) that is highly expressed at the BBB and transports drug into the brain. In this review, we summarized the strategies of crossing BBB, properties of EAAT3 and its natural substrates, Cys and its donors, and Cys donor-based brain-targeting prodrugs by referring to recent investigations. Moreover, the challenges that we are faced with and future research orientations were also addressed and proposed. It is hoped that present review will provide evidence for the pursuit of novel Cys donor-based brain-targeting prodrug.

## 1. Introduction

Currently, the aggravation of population aging trend has led to an increasing prevalence of neurodegenerative diseases, in particular of Alzheimer's disease (AD) and Parkinson's disease (PD) have brought heavy burdens to society and economy [1]. However, due to the existence of blood-brain barrier (BBB), practically 100% macromolecular and over 98% small-molecular drugs cannot enter the brain effectively, leading to most of the current available remedies far from impressive, and are unable to halt or delay the pathological processes [2]. BBB, the tightest physiologic barrier consisting mainly of endothelial cells linked by tight and adherens junctions, separates the blood circulation system from the central nervous system (CNS) [3]. Only a limited number of substances, e.g., most of lipophilic compounds with molecular weights < 400 ~ 500 Da, can cross the BBB via simple diffusion to reach efficacious concentrations in brain [4]. Under normal physiological conditions, BBB plays a key role in maintaining brain homeostasis, but when facing pathological state, it also strictly limits the entrance of therapeutic drugs into the brain. Thus, the search for novel brain-targeting methods still remains an ongoing concern.

One of the common features of neurodegenerative disorders is extensive evidence of oxidative stress, which is most likely to be associated with the progressive loss of specific neuronal cells [5]. Oxidative stress is defined as an imbalance between reactive oxygen species (ROS) generation and elimination [6]. The excessive ROS can oxidize proteins, lipids, and DNA to initiate neuronal cell death, which is the most common feature of neurodegenerative diseases, due to the fact that the brain is the most vulnerable organ to ROS in the body [7].

Glutathione (GSH), the storage form of cysteine (Cys), is a tripeptide compound widely distributed in virtually all cells. It actively participates in oxidation-reduction reactions, ROS elimination, gene expression, signal transduction, cell proliferation and apoptosis, immune response, cytokines production, as well as the synthesis of DNA and proteins [8, 9]. In CNS, GSH is pivotal for the prevention of cells from oxidative stress-mediated damages [10]. However, GSH levels in brain usually decline with aging owing to the increased formation of ROS, which may be the most robust risk factor for susceptibility to neurodegenerative disorders [11, 12].

**Figure 1:**
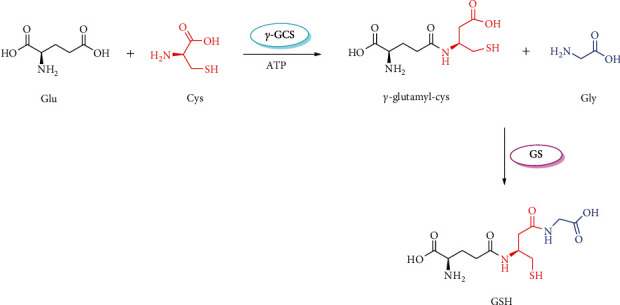
The biosynthesis of glutathione. Glu: glutamate; Cys: cysteine; *γ*-GCS: *γ*-glutamylcysteine synthetase; ATP: adenosine triphosphate; Gly: glycine; GS: glutathione synthetase; GSH: glutathione.

Endogenous GSH is synthesized in the cytoplasm of cells starting from glutamate (Glu), Cys, and glycine (Gly) by two enzymatic reactions involving the consumption of adenosine triphosphate (ATP). *γ*-Glutamylcysteine synthetase (*γ*-GCS) catalyzes the first reaction by combining Glu and Cys to produce *γ*-glutamyl-Cys, followed by reacting with Gly to yield GSH under the catalysis of GSH synthetase (GS) [13] (Figure 1). The first reaction mediated by *γ*-GCS is a rate-limiting step that regulates GSH synthesis via feedback inhibition of *γ*-GCS [10, 14]. Due to relatively higher intracellular concentrations, extracellular supplements of Glu and/or Gly usually fail to elevate GSH level, making Cys the rate-limiting substrate for neuronal GSH synthesis [15, 16]. It would provide proof that exogenous supplement of Cys potentiates the biosynthesis of GSH in brain. The excitatory amino acid transporter 3 (EAAT3, also known as EAAC1 in rodents) is extensively distributed in brain capillary endothelial cells, neurons, and astrocytes. Compared with other members of EAAT family, EAAT3 possesses higher activities [17] and mainly mediates the neuronal uptake of Cys [18], indicating that EAAT3 may play a predominant role in transporting Cys from blood into the brain.

The well-known free radical-scavenging capacities of Cys and its crucial role in enhancing brain GSH suggest that Cys-based prodrug may potentiate the efficacy of therapeutic drug against neurodegenerative diseases. Moreover, prodrug with Cys moiety could be recognized by EAAT3 to overcome the restriction resulting from the BBB to improve the brain bioavailability of therapeutic drug. Herein, we summarized the methods to overcome BBB, EAAT3 and its substrates, Cys and its donors, and Cys donor-based brain-targeting prodrugs by referring to recent studies. Then, we discussed the opportunities and challenges on this emerging brain-targeting drug delivery strategy for oxidative stress-associated brain disorders.

## 2. Methods to Overcome BBB

Generally, four different mechanisms, including simple diffusion, facilitated diffusion, simple diffusion via aqueous channels, and active transport via transporters, involve in the BBB crossing of substances. In addition, transcellular and paracellular routes exert influences on substances across BBB. Only hydrophobic substances can be mediated by transcellular diffusion to cross the phospholipid membrane of endothelial cells, and substances with small-molecular weights can cross the BBB mediated by paracellular diffusion due to the restriction of tight junctions [19]. Moreover, other mechanisms, such as adsorptive-mediated transcytosis, carrier-mediated transport, receptor-mediated transport, cell-mediated transport, and inhibition of efflux pumps, also participate in transport across the BBB for certain substances [20]. These transportation mechanisms provide clues for the development of novel methods to overcome BBB.

In the last several years, nanoparticle- (NP-) based drug delivery system has gained intense attentions as an emerging brain-targeting strategy. These NPs comprise micelles, liposomes, dendrimers, polymers, and inorganic NPs [21]. They can load and deliver therapeutic drugs to brain through opening the tight junctions between endothelial cells, transcytosis, endocytosis, and combination of several aforementioned mechanisms [19, 22]. Certainly, NPs have brought breakthroughs and hopes for overcoming BBB, but some methodological and safety issues limit their successful applications for disease treatment [23–25]. The ideal CNS-targeting strategies should be characterized by targeted transport of therapeutic drugs into the brain without changing their pharmacological activities and low amount of systemic distribution to avoid potential untoward effects. In this respect, the prodrug transported via specific transporter that is highly expressed at the BBB is deemed as a promising approach to enhance the brain uptake of small-molecular-weight drugs [26, 27]. A prodrug, an inactive form of drug, can be transformed into the pharmacologically active drug (parent drug) by enzymatic and/or chemical reactions to exert intended effects after administration. The prodrugs are usually designed to improve treatment outcomes relative to parent drugs, including optimizing pharmacokinetic properties, reducing side effects, and improving drug selectivity and targeting [28]. Approximately, 5%~7% of marketed drugs worldwide can be categorized as a kind of prodrug [29].

**Figure 2:**
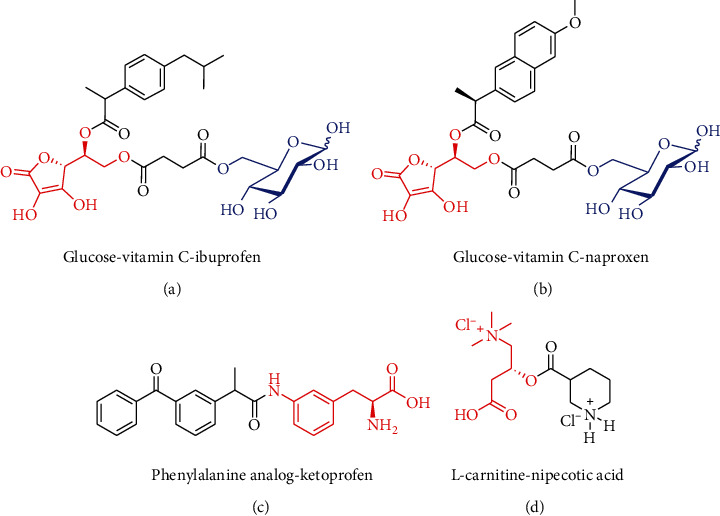
The selected cases of brain-targeting prodrugs transported via endogenous transporters at the BBB. (a) Ibuprofen prodrug transported via GLUT1 and SVCT2; (b) naproxen prodrug transported via GLUT1 and SVCT2; (c) ketoprofen prodrug transported via LAT1; and (d) nipecotic acid transported via OCTN2. GLUT1: glucose transporter 1; SVCT2: sodium-vitamin C transporter 2; LAT1: large neutral amino acid transporter 1; OCTN2: organic cation/carnitine transporter 2.

The transporters specifically and highly expressed at the BBB provide us with new concepts for the design of novel brain-targeting prodrugs, i.e., therapeutic drug is designed to be combined with an endogenous substrate or substrate analog of transporter via functional group to synthesize a prodrug, which is expected to be recognized and transported by specific transporter *in vivo* to cross the BBB. In the brain, the therapeutic drug is released from prodrug under enzymatic and/or nonenzymatic conditions [30].  Table 1 exhibits the common transporters highly expressed at the BBB. These transporters are served as vectors that are responsible for the transport of nutrients from peripheral blood into the brain. Many of them have been focused and successfully applied for the design of brain-targeting prodrugs. For example, a large number of studies have confirmed that regular consumption of nonsteroidal anti-inflammatory drugs (NSAIDs), such as ibuprofen, naproxen, and ketoprofen, could delay the onset of CNS disorders. However, the low-level BBB permeability hindered the applications of NSAIDs for CNS diseases [31]. Yue et al. modified ibuprofen with glucose and vitamin C to synthesize a dual-mediated prodrug (glucose-vitamin C-ibuprofen, Glu-VC-Ibu, Figure 2(a)), which was intended to cross the BBB via GLUT1 and SVCT2. The results showed that Glu-VC-Ibu experienced a slow degradation in blood after administration to the mice, while in the brain, the concentration of ibuprofen released from Glu-VC-Ibu was almost three times higher than that of ibuprofen at 45 min, suggesting that Glu-VC-Ibu could be a slow-releasing and long-acting prodrug [32]. Based on the similar strategy, Wang et al. designed and synthesized a dual brain-targeting naproxen prodrug (glucose-vitamin C-naproxen, Glu-VC-Nap, Figure 2(b)). The Glu-VC-Nap equally elicited better *in vivo* brain uptake properties and neuroprotective effects than those of naproxen [33]. Puris et al. combined ketoprofen with phenylalanine analog to develop a LAT1-utilizing prodrug (ketoprofen-phenylalanine analog, Ket-Phe, Figure 2(c)) and explored the transport mechanism across the BBB. The results indicated that (1) the intrabrain amount of Ket-Phe was approximately 16 times higher than that of ketoprofen; (2) LAT1 involved in the transport of Ket-Phe into the brain, but its protein expression and amino acid exchange were not altered; and (3) the overall amount of prodrug-released ketoprofen in brain was almost 11 times higher than that of ketoprofen [34]. In another case, nipecotic acid, an anticonvulsant drug with low-level brain uptake, was modified with *L*-carnitine to prepare a double prodrug (*L*-carnitine-nipecotic acid, Car-Nip, Figure 2(d)), which was expected to be recognized and transported by OCTN2. After injection for 30 min in mice, the intrabrain concentration of nipecotic acid released from Car-Nip was up to 600 nmol/g, but for nipecotic acid control, the intrabrain nipecotic acid was hardly to be detected. In pentylenetetrazole-induced convulsion of mice, Car-Nip exerted convulsion latency of 1021 ± 55 s, while the counterpart in nipecotic acid control was only 645 ± 50 s [35, 36].

From theory to practice, the above-mentioned selected cases have successfully confirmed the feasibility and rationality of brain-targeting prodrug transported via endogenous transporters at the BBB, providing us a novel way to overcome BBB. In addition, new drug development based on prodrug strategy possesses advantages of low rick, small cost, and short cycle compared with the de novo drug design, owing to the fact that prodrug design is focused on the structural modification of postmarket drug, which will be released from prodrug *in vivo* to elicit pharmacological actions, including both therapeutic and adverse effects that have been fully checked and recognized during the long-term clinical use.

## 3. EAAT3

Glutamate (Glu), one of the major excitatory neurotransmitters in CNS, mediates a lot of important physiological processes. Nevertheless, high accumulation of extracellular Glu could cause hyperactivity of glutamatergic system, leading to neuronal injury and the onset of several CNS disorders [54]. Excitatory amino acid transporters (EAATs) play a key role in the maintenance of Glu homostasis in the brain. They not only participate in the uptake of Glu from synaptic cleft into neural cells but also involve in the clearance of excessive Glu released by neurons [53]. EAATs encompass five different subtypes, designated as EAAT1~ 5 in humans, while in rodents, subtypes of EAAT1 ~ 3 are also called glutamate-aspartate transporter (GLAST, corresponding to human EAAT1), glutamate transporter 1 (GLT1, corresponding to human EAAT2), and excitatory amino acid carrier 1 (EAAC1, corresponding to human EAAT3), respectively. These EAATs share most of the common features but differ in affinities and transportation rates of substrates as well as characteristics of functional regions [55–57].

**Figure 3:**
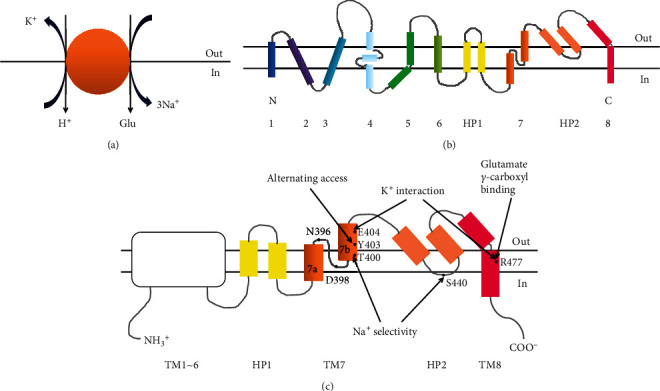
Schematic representation of the molecular properties of EAATs family. (a) Ion exchange and stoichiometry of glutamate transported by EAATs members; (b) transmembrane topology model of EAAT2; and (c) main functional regions and binding sites of EAAT2.

Recently, the properties and functions of EAAT3 have drawn extensive attentions, due to its brain-wide distribution and unique transport functions. The EAAT3 is found to be highly expressed on the small and large pyramidal neurons throughout the brain and is the only EAAT subtype that is responsible for the simultaneous transport of Glu and Cys. In contrast to other EAAT members, approximately 90% of the neuron Cys uptake from extracellular source is mediated by EAAT3. At the same time, recent researches have corroborated that deficiency of brain EAAT3 expression may result in a series of brain diseases, such as epilepsy, Parkinson's disease, Huntington's disease, ischemic stroke, and Alzheimer's disease [53, 58–61]. Like other EAAT members, the EAAT3 belongs to a sodium-dependent transporter. The uptake of Glu is driven by the cotransport of sodium ion (Na^+^), proton (H^+^), and potassium ion (K^+^) at a stoichiometric proportion of 3Na^+^/1H^+^/1 K^+^ [62, 63] (Figure 3(a)). The EAAT3 is encoded by gene *SLC1A1* and comprises 524 amino acid residues with molecular weight of 64 kDa [64–66]. Although the protein crystal structure of EAAT3 has not yet been disclosed currently, the transmembrane topology model of EAAT2 reported by Yernool et al. could be beneficial for the deep understanding of protein functions of EAAT family (Figures 3(b)~ 3(c): (1) EAAT2 consists of 8 membrane-spanning regions (TM1 ~ 8) and 2 helical hairpin regions (HP1 ~ 2), of which TM1 ~ 6 belong to conserved areas, and functional domains are mainly located near the C-terminal region; (2) Thr-400 and Ser-440 from HP2 are responsible for the coupling with Na^+^, while Tyr-403 and Glu-404 from TM7b are required for the interaction with K^+^; and (3) Arg-477 from TM8 is crucial for the interaction with the C-carboxyl group of substrates and is also important for the coupling with K^+^ [67, 68]. At present, there is no consensus regarding whether or not EAAT subtypes share certain common structures, but similarities in their amino acid sequences give a strong hint that universal structures could exist among different EAAT members, which deserve to be further explored. In the Arg-447 mutant of EAAT3 (corresponding to the Arg-477 of EAAT2), Glu transport is not observed, but Cys transport is normal, suggesting that Cys transport mediated by EAAT3 is not governed by Arg-447, which is, however, the key site that controls the binding of C-carboxyl group of substrates with EAAT2 (Arg-477) [68, 69]. Further investigation demonstrated that EAAT3 possesses equivalent affinity to Glu and Cys, and the affinity to Cys is 10 and 20-folds higher than that of EAAT1 and EAAT2, respectively. Moreover, Cys uptake mediated by EAAT3 belongs to unidirectional transport, which is not likely to cause the depletion of intracellular Cys [61, 70].

**Figure 4:**
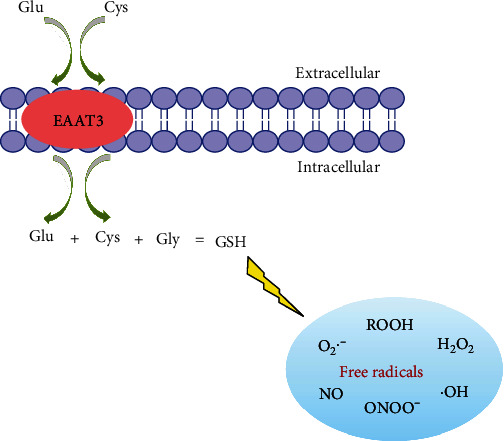
Schematic representation of the biosynthesis and free radical-scavenging capacities of GSH in brain.

As mentioned above, Cys is the rate-limiting substrate for GSH synthesis [71], thus the Cys uptake mediated by EAAT3 is of vital importance to GSH production in brain. The GSH is one of the endogenously generated antioxidants, particularly in the brain, and its level is significantly higher (2~3 mM) than that in peripheral blood (15 *μ*M) [72, 73]. Accordingly, oxidative stress induced by progressive GSH deficiency is usually considered to be one of the earliest biomarkers of aging, Parkinson's disease, and other neurodegenerative disorders [74]. Both Glu and Cys enter the brain by an EAAT3-mediated transport, followed by combination with Gly to synthesize GSH, which elicits scavenging capacity against free radicals, including hydroperoxides (ROOH), hydrogen peroxide (H_2_O_2_), hydroxy radical (^.^OH), nitric oxide (NO), hyperoxide (O^.-^_2_), and peroxynitrite (ONOO^−^) (Figure 4). In the EAAT3-deficient mice, when compared with normal control, brain GSH level was lower and the brain lesion caused by oxidative stress was greater. These aberrant changes can be attenuated and partially reversed by the administration of *N*-acetylcysteine, a precursor of Cys [75]. These results indicated that EAAT3 plays a particularly essential role in maintaining brain GSH level against oxidative stress. However, owing to the existence of sulfydryl group, Cys is often automatically oxidized to form cystine (Cys-Cys) in peripheral blood, leading to a lower uptake of Cys mediated by EAAT3 relative to that of Glu in the brain [76], which could be another trouble for the production of GSH in the brain. The occurrence and development of at least 100 diseases might be attributed to oxidative stress, especially for various neurodegenerative disorders [77, 78]. It is therefore important to exogenously supplement Cys beneficial for the yield of brain GSH and reduction of oxidative stress.

To sum up, EAAT3 possesses higher affinity to Cys than that of other EAAT members, and its transport of Cys could not be governed by the substrate carboxyl-binding site (Arg-447), suggesting that substrate specificity of EAAT3 might not be very rigid. Furthermore, Cys is the rate-limiting substrate for GSH synthesis, and delivery of Cys into the brain can boost brain GSH level to reduce oxidative stress and protect nerve. In theory, drugs with insufficient brain uptake could be considered to conjugate with Cys to give dual prodrug, which is expected to be recognized by EAAT3, then cross BBB into the brain, where it is metabolized by enzymes to release parent drug and Cys, thereby lifting the brain uptake of parent drug and indirectly increasing the brain GSH level.

## 4. Cys and Its Donors

The single dose of GSH as medication usually does not restore the *in vivo* GSH level, in some organs such as intestinal tract and liver, GSH can be biodegraded quickly, leading to an insufficient amount of delivery to the brain. Similarly, the consumption of Cys is also subjected to a poor recovery of GSH in the brain due to its relatively higher metabolic activity [79, 80]. It is therefore essential to replenish Cys in the form of precursors for the recovery of GSH level, particularly in the brain.

**Figure 5:**
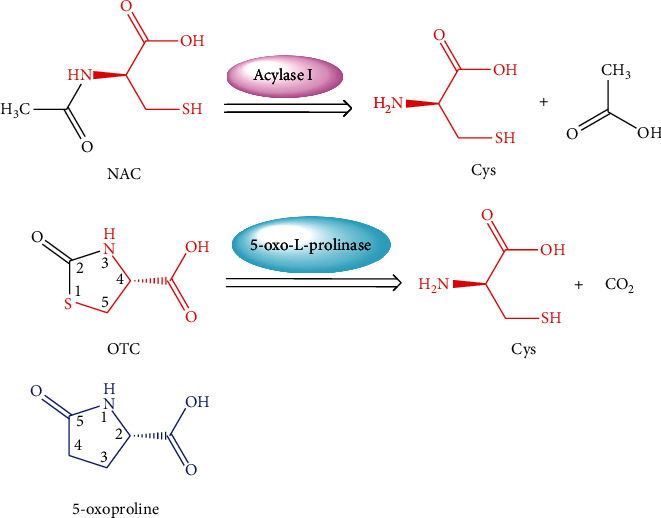
Chemical structures and metabolic pathways of NAC and OTC *in vivo*. NAC: *N*-acetylcysteine OTC: *L*-2-oxothiazolidine-4-carboxylic acid.

Up to now, several Cys donors, including glucose-cysteine [81], *L*-ribose-cysteine [82], 2-*N*-propylthiazolidine-4-carboxylic acid [83], *N*-acetylcysteine [84], 2-methylthiazolidine-4-carboxylic acid, 2-*N*-propylthiazolidine-4-carboxylic acid [85], *S*, *N*-diacetylcysteine monoethyl ester [86], *L*-2-oxothiazolidine-4-carboxylic acid [87], and 2-methyl-thiazolidine-2, 4-dicarboxylic acid [88], have been reported and developed. Among them, *N*-acetylcysteine (NAC) and *L*-2-oxothiazolidine-4-carboxylic acid (OTC) are the Cys donors that received extensive investigations and applications (Figure 5). Since the 1960s, NAC has been served as Cys donor for mucolytic remedy and acetaminophen poisoning. Since the 1980s, NAC has been also recommended as a relevant medication required in oxidative stress-related diseases. After being administered, in addition to exerting antioxidant capacity, NAC is metabolized by acylase I to release Cys (Figure 5), thereby promoting the biosynthesis of GSH [89, 90]. In the same way, OTC, also called as procysteine™, is another well-acknowledged Cys donor. Compared with NAC, OTC belongs to a more stable Cys donor that seals both the amino and the sulfydryl groups of Cys (Figure 5), avoiding of the spontaneous formation of disulfide linkage induced by free sulfydryl group [76]. *In vitro*, bioactivities of OTC are inferior to those of NAC [91], but *in vivo* or in cells that express 5-oxo-*L*-prolinase, OTC can be converted into Cys to elicit relevant bioactivities, due to its extremely similar structure with 5-oxo-proline, a natural substrate of 5-oxo-*L*-prolinase (Figure 5) [92]. OTC shows favorable safety properties [93] as well as diverse physiological and pharmacological effects, such as GSH-boosting capability, antioxidant and anti-inflammatory functions, hepatic and cardiac protections, as well as anti-ischemic stroke effect (Table 2). To a greater or lesser extent, these beneficial functions may be associated with the direct antioxidation of Cys released from OTC [94, 95] and the secondary cytoprotection of increased GSH against free radicals induced by oxidative stress [96]. As stated above, OTC is more stable than NAC, can resist oxidation during the *in vivo* distribution, conferring it promising potentials to cross BBB into the brain. In fact, consumption of OTC is indeed able to increase brain concentrations of Cys and GSH [97, 98] and has been proposed as a remedy strategy for combating oxidative stress in neurodegeneration [99, 100].

## 5. Cys Donor-Based Brain-Targeting Prodrug

**Figure 6:**
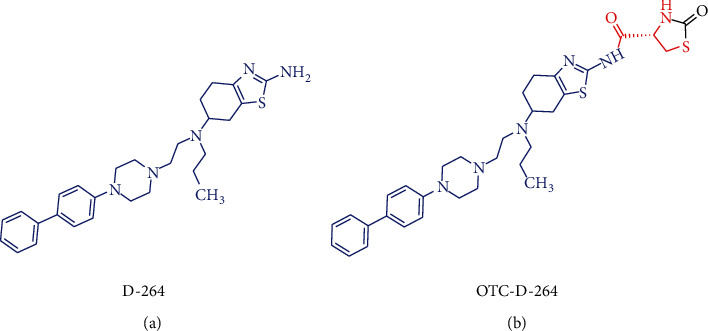
Chemical structures of D-264 (a) and its OTC based prodrug OTC-D-264 (b).

The stable pharmacokinetic properties and prominent capacities against oxidative stress are likely to make OTC a desirable Cys donor-based prodrug carrier that could deliver both Cys and parent drug into the brain, for the purpose of antioxidation and improving brain uptake of therapeutic agent. Moreover, the free carboxyl group of OTC provides a binding site for prodrug modification (Figure 5). Compound D-264 (Figure 6(a)), a potent D3 receptor-preferring agonist, exerts notable neuroprotection, neurotrophin-like activity, and hypolocomotion reversion, exhibiting great latent capacity for the precaution and treatment of Parkinson's disease [123–125]. However, D-264 seems to be less bioavailable in brain, which has hindered its further development and application [125]. Dholkawala et al. [126] designed and synthesized an OTC-based D-264 prodrug (OTC-D-264, Figure 6(b)), with the aim to enhance the BBB crossing efficacy and brain bioavailability of D-264, as well as to replenish antioxidant (Cys) and source for GSH synthesis in the brain. The designed dual prodrug OTC-D-264 was hypothesized to be recognized by EAATs or LAT1 expressed at the BBB and to be transported into the brain. The results showed that (1) OTC-D-264 was stable in rat plasma, the degradation rate (DR) was 9% within 24 h and was 21% within 48 h; (2) OTC-D-264 also experienced relatively slower degradation in the rat brain tissue but faster than that in rat plasma, DR was 86% within 48 h; and (3) the total concentrations of D264 and OTC-D-264 in the rat brain were 7.5-folds higher than those of D264 after 2 h, while after 4 h, total concentrations in brain were still 5.7-folds higher than those of D264. These favorable results indicated that after being dosed, OTC-D-264 is likely to be delivered to the brain without any degradation within the period of distribution in peripheral blood and could bring D-264 together to cross BBB into the brain, where it could be converted into D-264 in a slow-release manner, which might be a well-received property for the therapy of Parkinson's disease [127]. This innovative application has successfully corroborated the feasibility of OTC for improving brain uptake of therapeutic medicines. However, some unsettled issues are still pending: (1) Penetration mechanism of OTC-D-264 crossing the BBB. Although the positive effect of OTC on increasing the BBB permeability of D-264 has been confirmed, the transport mechanism is still unclear, simple diffusion or transport mediated by transporters? As mentioned earlier, EAAT3 has high affinity to Cys, and its transport of Cys could not be controlled by Arg-447 (key binding site for carboxyl group of substrates), indicating that the substrate specificities of EAAT3 might not be very strict [61, 68]. Therefore, whether or not, OTC could be equally recognized by EAAT3 as Cys. Moreover, what are the structural characteristics of EAAT3 substrates? (2) Potentials of antioxidant therapy in the brain. Single administration of OTC can increase brain levels of Cys and GSH to fight against oxidative stress-related neurodegenerative disorders [97–100]. However, when OTC is conjugated with drugs, how about its contribution to combat oxidative stress? Given that the parent drug is prescribed in a low dose, whether or not OTC-conjugated prodrug can enhance GSH level and antioxidant capacity. What is the proper molar ratio range between parent drug and OTC? How long is long enough for the medication? What is the proper administration route (oral or parenteral route)? Even the proper pharmaceutical dosage form should be also considered. (3) Based on OTC modification, whether or not novel prodrug carriers that possess Cys-donor properties can be developed.

**Figure 7:**
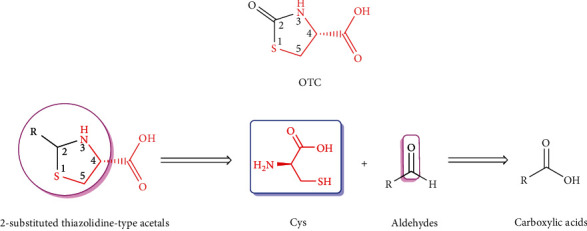
Design mentality of 2-substituted thiazolidine-type derivatives based on OTC as potential Cys donors and prodrug carriers.

**Figure 8:**
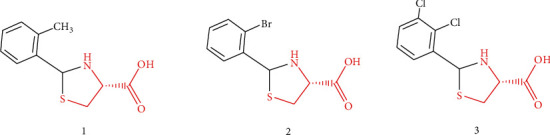
Chemical structures of 2-phenyl-thiazolidine-4-carboxylic acid derivatives that possess capabilities of releasing Cys in methanol.

The proof-of-concept that Cys donors should entirely release Cys without any structural changes guides us toward the rational design of OTC-based prodrug carrier. Through the structural analysis of OTC (Figure 7), it can be seen that (1) 4-Carboxyl group is not only a part of Cys but also the conjugation site with drug, thus it cannot be modified; (2) Similarly, 1-S, 3-*N*, and 5-C are parts of Cys, they also cannot be replaced by bioisosteres; and (3) 2-Carbonyl group is none of Cys, thus it is the right site that can be substituted by various substituents. The amino and sulfydryl groups of Cys belong to strong nucleophiles, which can easily react with the carbonyl groups of aldehydes to give thiazolidine-type derivatives, thereby constructing the 2-substituted OTC-based prodrug carriers (Figure 7). *In vivo*, these thiazolidine-type derivatives are proposed to be metabolized into Cys and aldehydes, and the resulting aldehydes can be subsequently oxidized to carboxylic acids that are easy to be eliminated from the body [128, 129] (Figure 7). Actually, Önen Bayram et al. [130] has preliminarily verified the Cys-release potentials of some thiazolidine-type derivatives. In their work, Cys was conjugated with aldehydes that possess different phenyl rings to synthesize serials of 2-phenyl-thiazolidine-4-carboxylic acid derivatives, whose stabilities and antioxidant capacities were then assessed. It was concluded that the Cys-release capacity and antioxidant efficacy partially rely on the substituents of 2-phenyl rings, and the characteristics of the aldehydes that are combined with Cys should be considered when designing prodrug carriers, owing to the fact that the structures of aldehydes can influence the Cys-release patterns of the resulting thiazolidines.  Figure 8 illustrated the chemical structures capable of releasing Cys in methanol.

Research on thiazolidine-type derivatives as Cys donors is only in its infancy, with regard to the potential of becoming Cys donor-based brain-targeting prodrug carriers (CDBPCs), additional investigations need to be deepened. Ideal CDBPCs should be stable during the distribution in peripheral blood and carry parent drug to cross BBB into the brain, where it can be metabolized to release both parent drug and Cys [126, 131]. Therefore, in the next study, for the pursuit of novel CDBPCs, researchers should focus on the relationships between the structures of thiazolidine-type derivatives and their stabilities in the peripheral blood and brain, abilities to penetrate BBB, as well as capacities of antioxidation and/or GSH-boosting capability. Moreover, the underlying mechanism on BBB permeability, particularly the interaction with EAAT3, should be also paid attention to pick up information on whether thiazolidine-type derivatives access to the opportunity of being recognized by EAAT3 and the structure-EAAT3 affinity/transport relationships.

## 6. Discussion and Conclusion

How to overcome BBB to ameliorate brain bioavailability of therapeutic agents is a challenging concern. There are various means of improving drug bioavailability to enable them more powerful. Thereinto, prodrug strategy seems to be a desirable approach without changing the structure and activity of parent drug. One of the most feasible and reliable methods for delivering drugs with low-level BBB permeability is to conceive a prodrug that potentiates parent drug to cross BBB into the brain [132]. Carrier-mediated prodrug (CMP) is crucial in transporting active agents into the brain. With the advance of understanding in transmembrane transport mechanisms of CMP and the involved transporters, particularly which are highly expressed at the BBB (Table 1), it is practicable to design the carriers of CMP by referencing the endogenous substrates of these transporters. Furthermore, it is also documented that brain uptake of Cys is chiefly mediated by EAAT3, substrate specificities of which might not be very rigid [53, 61, 68], suggesting that Cys analogs could be equally recognized by EAAT3. In this regard, Cys and its analogs have a hope of being applied as carriers of brain-targeting CMP. Meanwhile, the universally-known antioxidant capacity of Cys and its critical role in GSH synthesis are beneficial for the precaution and remedy of neurodegenerative diseases [12, 74, 96]. It is therefore important to develop Cys and/or its analogs into the carriers of CMP, thereby improving the brain bioavailability of parent drug and elevating the capacity against free radicals induced by oxidative stress. The OTC is a promising Cys donor that has been successfully used to potentiate anti-Parkinson's candidate drug across BBB into the brain [126]. Afterwards, some thiazolidine-type derivatives were designed and synthesized based on OTC, and three of them have been verified to possess capabilities of releasing Cys [130].

The OTC and its thiazolidine-type derivatives are likely to give us opportunities to access an emerging Cys donor-based brain-targeting prodrug carrier. However, we are also faced with some challenges, such as the penetration mechanism crossing BBB, structure-stability- antioxidation-brain bioavailability relationships, characteristics of EAAT3 substrates, and the structure-EAAT3 affinity/transport relationships. The disclosure of these scientific questions will greatly promote the research and development of novel Cys donor-based brain-targeting prodrug carrier.

## Figures and Tables

**Table 1:** Transporters highly expressed at the BBB.

Transporter	BBB localization	Endogenous substrate	Reference
Glucose transporter 1 (GLUT1)	Luminal and abluminal membranes of capillary endothelial cells	Glucose and some other hexoses	[37–39]
Sodium-vitamin C transporter 2 (SVCT2)	Surface of choroid plexus epithelium cells	Vitamin C	[31, 40]
Large neutral amino acid transporter 1 (LAT1)	Luminal and abluminal membranes of capillary endothelial cells and brain parenchyma cells	Large and branched neutral amino acids	[41–43]
Cationic amino acid transporter 1 (CAT1)	Astrocytes, oligodendrocytes, endothelial cells, and neurons	Arginine, lysine, and ornithine	[44, 45]
Monocarboxylic acid transporter 1 (MCT1)	Luminal and abluminal membranes of capillary endothelial cells	Lactic and pyruvic acids	[46, 47]
Organic cation/carnitine transporter 2 (OCTN2)	Basolateral membrane and cytoplasmic region	*L*-carnitine	[36, 48]
Concentrative nucleoside transporter 2 (CNT2)	Brain capillary endothelium	Adenosine	[49]
Choline transporters (CHTs)	Cholinergic neurons	Choline	[50, 51]
Excitatory amino acid transporters (EAATs)	Abluminal membranes of endothelial cells, astrocytes, and neurons	Glutamate and cysteine	[52, 53]

**Table 2:** Physiological and pharmacological effects of OTC.

Effect	Subject/model	Dosage	Results	Reference
GSH-boosting capability	*γ*-Glutamyltranspeptidase-deficient knockout mice	8.0 mg/mL drinking water, dietary supplementation for 5 consecutive weeks	Replenish GSH pool and provide protection from apoptosis	[101]
	Protein-malnourished mice	59.5 mmol/kg BW, dietary supplementation for 1 week	Restore tissue GSH level and redox status	[102]
	Asymptomatic patients infected with human immunodeficiency virus	Single dose of 500 mg, 1500 mg, and 4500 mg, p.o., respectively; 14 days later, followed by 500 mg, 1500 mg, and 3000 mg, p.o., three times per day for 28 consecutive days, respectively	Single-dose administration: plasma levels of OTC can be measured at all doses; four-week administration three times daily: increase whole blood GSH at doses of 1500 mg and 3000 mg	[103]
	Peritoneal dialysis patients	500 mg, p.o., three times per day for 14 consecutive days	Increase whole-blood GSH	[104]
	SAA-deficient aged mice	0.5% relative to diet, dietary supplementation for 4 consecutive weeks	Correct aging-associated differences in hepatic GSH and GSH/GSSG ratio via upregulating nSMase-2 expression and via increasing ceramide level	[105]

Antioxidant and anti-inflammatory properties	*In vitro*: TNF-*α*-stimulated ARPE-19 cells; *In vivo*: DKO rd8 mice	*In vitro*: 0.1 mM, 0.5 mM, and 1.0 mM; *In vivo*: 10 mg/mL drinking water, dietary supplementation for 5 consecutive months	Inhibit IL-6, CCL2, and other biomarkers of inflammation via agonizing anti-inflammatory GPR109A and transportable substrates of SMCT1	[106]

Hepatic protection	Chronic ethanol-induced liver injury in rats	500 mg/kg BW, dietary supplementation for 4 consecutive weeks	Decrease AST, necrosis, inflammation, superoxide production, TNF-*α* and NF-*κ*B; Increase circulating GSH levels to inhibit the activation of Kupffer cells via glycine-gated chloride channel	[107]
	TAA-induced hepatic fibrosis in rats	80 mg/kg BW and 160 mg/kg BW, i.p., 30 min ahead TAA injection, three times per week for 8 consecutive weeks	Restore antioxidative system by upregulating Nrf2 to improve liver function parameters, ameliorate liver fibrosis, and decrease hepatic MDA	[108]

Anticataract effect	ACP-induced cataractous mice	At 0 h, 2.7 mmol/kg BW (OTC), i.p.; At 0.75 h, 3.0 mmol/kg (ACP), i.p.; At 1.25 h, 1.8 mmol/kg BW(OTC), i.p.	Prevent cataract formation via maintaining hepatic GSH homeostasis	[109]

Cardiac protection	Endotoxin-induced ventricular dysfunction in rabbits	2.4 g/kg BW, s.c., hypodermic injection, 24 h before experiment, three times at 4-h intervals	Prevent early decrease in ventricular contractility via increasing myocardial GSH	[110]
	Patients with coronary artery disease	Single dose of 4.5 g, p.o.	Reverse endothelial dysfunction by augmenting intracellular GSH and improving flow-mediated dilation	[111]

Antiperitonitis effect	LPS-induced peritonitis in rats	Acute experiment: 5 *μ*g/mL (LPS) plus 5 mmol/L (OTC), i.p.; Pretreatment: 0.1% drinking water, dietary supplementation for 10 consecutive days before infusion of LPS; Chronic experiment: on days 8, 9, and 10, 5 *μ*g/mL (LPS) plus 5 mmol/L (OTC), i.p., respectively	Acute experiment: prevent the decrease of cellular GSH and the increase of dialysate cell count; Pretreatment: slow the permeability to proteins; Chronic experiment: prevent peritoneal thickening and neovascularization	[112]

Anti-HIV-1 activity	MDM and lymphocytes as well as chronically HIV-1-infected MDM cultures	Proliferation assay: 5 mM to 30 mM; HIV-1 RT assay: 20 mM; Assay for antiviral effect: 5 mM to 30 mM	Suppress HIV-1 expression, RT activity, and virus replication without cytotoxicity	[113]

Nephroprotection	Cisplatin-induced nephrotoxicity in rats	150 mg/kg BW, i.g., once daily for 7 consecutive days	Ameliorate histopathological and biochemical indices of nephrotoxicity via increasing SOD and GSH	[114]
	Cisplatin-induced nephrotoxicity in mice	80 mg/kg BW, i.g., once daily for 3 consecutive days	Decrease the production of ROS, translocation of NF-*κ*B p65 subunit into nucleus, infiltration of macrophages into renal tissue, and expression of ICAM-1, MCP-1, and caspase 3	[115]

Antidiabetic effect	*In vitro*: pancreatic islet cells; *In vivo*: C57BL/KsJ-*db/db* mice	*In vitro*: 1 mM; *In vivo*: 8 mg/kg BW to 80 mg/kg BW, i.g., twice daily for 4 consecutive weeks	Ameliorate glucose tolerance by heightening insulin secretion via CD38/cADPR/Ca^2+^ signaling pathway	[116]

Gastric protection	Ethanol-induced gastric lesions in rats	100 mg/kg BW, 200 mg/kg BW, and 400 mg/kg, i.p., for gastric secretion study; i.g., for antiulcer study	Reduce the acidity and volume of gastric secretion, attenuate the formation of gastric lesion, and protect the gastric mucosa against gastric wall mucus depletion, NP-SH, and MPO via inhibiting neutrophils and replenishing GSH	[117]

Antiasthmatic effect	OVA-induced allergic asthma in mice	40 mg/kg BW, 80 mg/kg BW, and 160 mg/kg per day, i.p., 4 times daily on days 21 to 24	Decrease airway hyperresponsiveness, bronchial inflammation, ROS production, IL-4, IL-5, IL-13, eosinophil cationic protein, ICAM-1, VCAM-1, RANTES, eotaxin, NF-*κ*B, and VEGF	[118, 119]

Antitumor activity	*In vitro*: B16F10 melanoma cells; *In vivo*: B16F10-induced multiple liver metastases in mice	*In vitro*: 1 mM; *In vivo*: 2.5 mmol/kg BW, i.p., 2 h before and 30 min after CY treatment on days 3, 6 and 8 (OTC + CY), daily from days 4 to 7 (OTC + IL − 2)	*In vitro*: Antagonise the growth-promoting effects induced by IL-2; *In vivo*: show antitumor activity and increase life span	[120]

Precaution of cerebral microvessel thrombosis	DEP-induced cerebral microvessel thrombosis in mice	80 mg/kg BW, i.p., 24 h and 1 h ahead intratracheal instillation of DEP	Abolish DEP-induced macrophage and neutrophil influx and the increased TEAC; protect DEP-induced lung inflammation; and reverse the decreased TEAC, shortened bleeding time, and thrombotic effect of DEP in pial cerebral venules through balancing oxidant-antioxidant status	[121]

Antiischemic stroke effect	MCAO in mice	50 mg/kg BW, 100 mg/kg BW, and 150 mg/kg BW, i.v., tail vein injection, 1 h before or after MCAO; 100 mg/kg BW, i.v., tail vein injection, 3 h or 6 h after MCAO	Reduce brain infarct injury and improve behavioral outcomes; increase GSH; decrease superoxides, neuroinflammation and oxidized proteins; and restore Ubqln1 and conjugated protein	[122]

Abbreviations: GSH: glutathione; p.o.: oral adminstration; BW: body weight; OTC: *L*-2-oxothiazolidine-4-carboxylic acid; SAA: sulfur-containing amino acid; nSMase-2: neutral Smase-2; GSSG: oxidized glutathione; ARPE-19: human retinal pigment epithelial cells; DKO rd8: *Ccl2^−/−^/Cx3cr1^−/−^* mice on rd8 mutation; CCL2: CC-chemokine ligand 2; GPR109A: G-protein coupled receptor 109A; SMCT1: sodium-coupled monocarboxylate transporter 1; AST: glutamic-oxalacetic transaminase; TNF-*α*: tumor necrosis factor-*α*; IL-6: interleukin-6; NF-*κ*B: nuclear factor-*κ*B; i.p.: intraperitoneal injection; ACP: acetarninophen; TAA: thioacetamide; Nrf2: nuclear factor erythroid 2-related factor 2; s.c.: subcutaneous injection; LPS: lipopolysaccharide; HIV-1: human immunodeficiency virus type 1; MDM: monocyte-derived macrophages; RT: reverse transcriptase; i.g.: intragastric administration; SOD: superoxide dismutase; CD38: cluster of differentiation 38; cADPR: cyclic adenosine diphosphoribose; Ca^2+^: calcium ion; ROS: reactive oxygen species; NP-SH: nonprotein sulfhydryls; MPO: myeloperoxidase; OVA: ovalbumin; IL-4: interleukin-4; IL-5: interleukin-5; IL-13: interleukin-13; ICAM-1: intercellular cell adhesion molecule-1; VCAM-1: vascular cell adhesion molecule-1; RANTES: regulated on activation, normal T cell expressed and secreted; VEGF: vascular endothlial growth factor; B16F10: murine B16 melanoma cell line; CY: cyclophosphamide; IL-2: interleukin-2; DEF: diesel exhaust particles; TEAC: Trolox equivalent antioxidant capacity; MCAO: middle cerebral artery occlusion; i.v.: intravenous injection.
